# Effect of an isoenergetic traditional Mediterranean diet on apolipoprotein A-I kinetic in men with metabolic syndrome

**DOI:** 10.1186/1475-2891-12-76

**Published:** 2013-06-07

**Authors:** Caroline Richard, Patrick Couture, Sophie Desroches, Alice H Lichtenstein, Benoît Lamarche

**Affiliations:** 1 Institute of Nutrition and Functional Foods, Laval University, 2440, boul. Hochelaga, Québec (Qc), G1V 0A6, Canada; 2 Lipid Research Center, CHUQ Research Center, Quebec, Canada; 3 Cardiovascular Nutrition Laboratory, Tufts University, Boston, MA, USA

**Keywords:** Diet, Apolipoproteins, Lipoproteins, Obesity, Metabolism

## Abstract

**Background:**

The impact of the Mediterranean diet (MedDiet) on high-density lipoprotein (HDL) kinetics has not been studied to date. The objective of this study was therefore to investigate the effect of the MedDiet in the absence of changes in body weight on apolipoprotein (apo) A-I kinetic in men with metabolic syndrome (MetS).

**Methods:**

Twenty-six men with MetS (NCEP-ATP III) were recruited from the general community. In this fixed sequence study, participants’ diet was first standardized to a control diet reflecting current averages in macronutrient intake in North American men, with all foods and beverages provided under isoenergetic conditions for 5 weeks. Participants were then fed an isoenergetic MedDiet over a subsequent period of 5 weeks to maintain their weight constant. During the last week of each diet, participants received a single bolus dose of [5,5,5-^2^H_3_] _L_-leucine and fasting blood samples were collected at predetermined time points. ApoA-I kinetic was determined by multicompartmental modeling using isotopic enrichment data over time. Data were analyses using MIXED models.

**Results:**

The response of HDL-cholesterol (C) to MedDiet was heterogeneous, such that there was no mean change compared with the control diet. Plasma apoA-I concentration (−3.9%) and pool size (−5.3%, both *P* < 0.05) were significantly lower after MedDiet and apoA-I production rate tended to be reduced (−5.7%, *P* = 0.07) with no change in apoA-I fractional catabolic rate (FCR, -1.6%, *P* = 0.64). Participants among whom HDL-C concentrations were increased with MedDiet (responders: mean ∆HDL-C: +9.9 ± 3.2%, N = 11) showed significantly greater reductions in apoA-I FCR and in apoB and very-low-density lipoprotein-triglycerides (VLDL-TG) concentrations (all *P* < 0.04) than those among whom HDL-C levels were reduced after the MedDiet (non-responders: mean ∆HDL-C: -12.0 ± 3.9%, N = 8). Correlation analysis revealed that only variations in apoA-I FCR (*r* = -0.48, *P* = 0.01) and in plasma VLDL-TG (r = −0.45, *P* = 0.03) concentrations were correlated with the individual HDL-C response to the MedDiet.

**Conclusions:**

Data from this controlled feeding study suggest that the heterogeneous response of HDL-C to MedDiet, in the absence of important weight loss, is primarily related to individual variations in apoA-I FCR and in plasma VLDL-TG concentrations.

**Trial registration:**

ClinicalTrial.gov registration number: NCT00988650

## Background

Low plasma high-density lipoprotein-cholesterol (HDL-C) and high plasma triglyceride (TG) concentrations are two diagnostic criteria of metabolic syndrome (MetS) [1]. Over-secretion of very-low-density lipoprotein-apolipoproteinB (VLDL-apoB) and accelerated clearance of HDL particles appear to be the primary mechanisms sustaining the high TG/low HDL phenotype in MetS [2].

Previous studies have demonstrated that when body weight is maintained constant, diets low in saturated fat and high in carbohydrates (CHO) have HDL-C lowering and TG raising effects [3]. On the other hand, several epidemiological studies have shown that adherence to Mediterranean type diet (MedDiet), which is characterized among other factors by a high consumption of monounsaturated fatty acids (MUFA) and low intake of saturated and trans fat, is associated with a reduced risk of overall mortality and death from cardiovascular disease [4]. However, the extent to which this is attributable to beneficial changes in HDL concentrations and function with MedDiet is unknown. A recent meta-analysis of 50 studies revealed that adherence to the MedDiet was associated with significant reductions in body weight and waist circumference [5]. Thus, it is not clear if the favorable increase in plasma HDL-C concentrations often seen with MedDiet is due to differences in diet composition *per se* or to concurrent reduction in body weight as well. A better understanding of how HDL metabolism is modified in response to MedDiet, *per se*, is crucial to help identify optimal dietary interventions for low HDL-C concentration management in high-risk individuals.

The objective of this study was to investigate the impact of the MedDiet, in the absence of weight change, on apolipoprotein (apo) A-I kinetics in men with MetS. We hypothesized that in contrast to prior data having documented the combined effect of the MedDiet and weight loss, short-term consumption of a traditional MedDiet in the absence of weight loss has no impact on the catabolic rate of apoA-I and thus on plasma HDL-C concentrations.

## Methods

### Population and study design

Details of the study design have been previously described [6]. Briefly, 26 men (18 to 65 years) diagnosed with the MetS (NCEP-ATP III [1]), and who did not smoke, with no history of coronary heart disease (CHD) or type 2 diabetes, and not using lipid-lowering or anti-hypertensive medication were recruited for the study. For inclusion in the study, men also had to have a stable weight for at least 6 months prior to the start of the study, not use vitamin supplements or natural health products, and have no aversion to specific components of the MedDiet. Study procedures were approved by the Research Ethics Committee of Laval University and written informed consent was obtained from all participants prior to be enrolled in the study.

### Isoenergetic experimental diets

Participants’ diet was first standardized to a control diet reflecting current averages in macronutrient intake in North American men [7]. Food was provided in isoenergetic conditions over a 5-week period to maintain body weight constant. This controlled feeding period was included in the protocol to minimize inter-individual variations attributed to each participant’s usual diet. Participants were then provided with a MedDiet (5 weeks) that was formulated to be concordant with characteristics of the traditional Mediterranean eating pattern again in isoenergetic conditions to maintain body weight constant [8]. Seven-day menus and daily servings of various food categories for the control diet and the MedDiet were developed for the study and have been described previously [6]. Mean nutritional composition of the control diet and the MedDiet are presented in Table 1.

**Table 1:** **Mean nutritional composition of the control diet and the MedDiet**^***a***^

**Nutrients**^***b***^	**Control diet**		**MedDiet**		***P***^***c***^
**Energy, kJ**	13179 ± 1936	-	13270 ± 1856	-	0.506
**Lipids, g/d (%)**	119.0 ± 17.5	(34.0%)	112.7 ± 15.8	(32.0%)	<0.001
**SFA, g/d (%)**	45.5 ± 6.7	(13.0%)	23.7 ± 3.3	(6.7%)	<0.001
**MUFA, g/d (%)**	46.0 ± 6.8	(13.2%)	63.8 ± 8.9	(18.1%)	<0.001
**PUFA, g/d (%)**	18.2 ± 2.7	(5.2%)	16.7 ± 2.3	(4.7%)	<0.001
**TFA, g/d (%)**	7.0 ± 1.0	(2.0%)	1.2 ± 0.2	(0.3%)	<0.001
**Cholesterol, mg/d**	414.1 ± 60.8	-	367.3 ± 51.4	-	<0.001
**Carbohydrates, g/d (%)**	380.9 ± 56.0	(48.4%)	396.4 ± 55.5	(50.0%)	<0.001
**Total fibers, g/d**	25.2 ± 3.7	-	53.6 ± 7.5	-	<0.001
**Soluble fibers, g/d**	9.2 ± 1.4	-	15.4 ± 2.2	-	<0.001
**Proteins, g/d (%)**	133.8 ± 19.7	(17.0%)	134.7 ± 18.8	(17.0%)	0.525
**Alcohol, g/d (%)**	9.0 ± 1.3	(2.0%)	22.7 ± 3.2	(5.0%)	<0.001
**Sodium, mg/d**	4406 ± 647	-	3853 ± 539	-	<0.001

^*a*^ Values are presented as mean ± SD or percentage of daily energy intake.

^*b*^ SFA, saturated fatty acids; MUFA, monounsaturated fatty acids; PUFA, polyunsaturated fatty acids; TFA, trans fatty acids.

^*c*^*P* value from the main effect of diet in the Mixed model (n = 26) for data expressed in g or mg/d. There is no variation in nutrient intake expressed in% of calories because all subjects received the same diet regimen based on a 2500 kcal/d menu.

All meals, foods and beverages including red wine were provided to participants at the clinical investigation unit (CIU) of the Institute of Nutrition and Functional Foods (INAF). For most men, lunch (40% of daily energy intake) was eaten at the CIU and dinners and breakfasts at home. Men were instructed to consume only the meals provided and to report any deviation from the prescribed protocol on checklists. The use of vitamin supplements, anti-inflammatory medications (NSAIDs) and natural health products was strictly forbidden during the entire experimental period. Subjects were instructed to maintain their usual physical activity level except for the 3 days that preceded blood sampling periods, during which they were asked to refrain from intense physical exercise.

### Kinetic protocol

All participants underwent a kinetic study during the last week of the control diet and the MedDiet. On each occasions after a 12-h fast, participants received a single i.v. bolus of [5,5,5-^2^H_3_] _L_-leucine (11 mg/kg) and fasting blood samples (20 mL) were collected at predetermined time points over a 96 hours period (0, 0.5, 1, 2, 4, 6, 8, 10 h). Additional twelve-hour fasting blood samples were collected in the morning of the next 4 subsequent days (24, 48, 72, 96 h). Participants remained on the study diets for the duration of the kinetic study (5 days).

### Plasma lipids and lipoproteins assessment

Plasma lipids were measured enzymatically on a Roche/Hitachi Modular using Roche Diagnostics reagents (Roche diagnostics GmbH, Mannheim, Germany) according to standardized procedures [9]. The cholesterol and triglyceride content of the HDL_2_ and HDL_3_ subfractions was determined after sequential precipitation with dextran sulfate as previously described [10]. Plasma apoA-I concentrations were measured by nephelometry (Dade Behring, Mississauga, Ontario, Canada). Fasting blood glucose concentrations were determined by the hexokinase-glucose-6-phosphate dehydrogenase method [11] and fasting insulin concentrations by radioimmunoassay [12].

### Quantification and isotopic enrichment of apolipoprotein A-I

ApoA-I in the d < 1.25 g/ml plasma fraction was obtained by ultracentrifugation using a Beckman 50.4ti Rotor. Samples were then dialyzed overnight in a NaCl-Tris-EDTA buffer. Following a cysteamine treatment for 4 h at 37°C, samples were delipidated according to standardized procedures [13]. ApoA-I was isolated by isoelectric focusing (IEF) on a polyacrylamide-urea gels and bands were excised. Bands containing apoA-I were then hydrolysed for 24 hours at 110°C using 6 N HCl, dried and derivatized. The isotopic enrichment (%) was determined by a gas chromatograph-mass spectrometer (GC-MS; GC 6890 N, MS 5973 N, Agilent Technologies, Palo Alto, CA).

### Kinetic modeling

**Figure 1:**
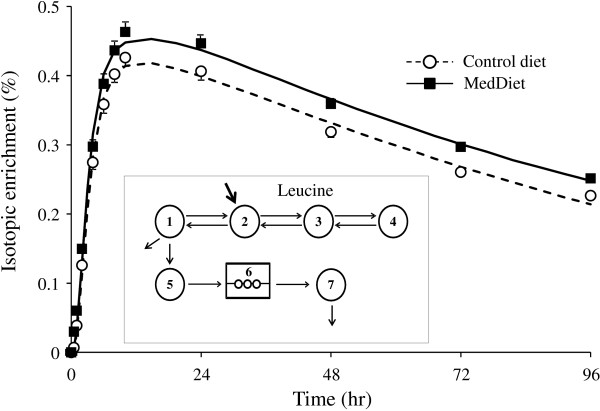
**Isotopic enrichment over time and multicompartmental model used to derive apolipoprotein A-I (apoAI) intravascular kinetic.** Mean isotopic enrichment over time of plasma apoA-I for the 26 men with MetS (symbols), model-predicted values (lines) and multicompartmental model used to determined kinetic parameters of apoA-I (insert). Compartments 1–4 reflect the kinetic of plasma leucine. Compartment 5 represents the intracellular pool of leucine (hepatic and other tissues) from which apoAI is synthesized and appears into the circulation (compartment 7) after a delay (compartment 6).

ApoA-I fractional catabolic rate (FCR) was determined by multicompartmental modeling of the isotopic enrichment data over time using the SAAM II software (University of Washington, Department of Bioengineering, Seattle, WA). Figure 1 shows the isotopic enrichment (%) over time and the multicompartmental model from which kinetic parameters are derived. Compartments 1–4 reflect the kinetic of plasma leucine. Compartment 5 represents the intracellular pool of leucine (hepatic and other tissues) from which apoAI is synthesized and appears into the circulation (compartment 7) after a delay (compartment 6). Fractional catabolic rate (pools/day) of plasma apoA-I was obtained from the best fit of isotopic enrichment of apoA-I over time to the model. Pool size (PS) of apoA-I was estimated by multiplying plasma apoA-I concentrations by plasma volume (estimated at 0.045 l/kg body weight) [14]. The production rate (PR in mg/kg/d) of apoA-I was calculated by multiplying the FCR by the PS of apoA-I, and correcting for body weight.

### Statistical analysis

Data are reported as mean ± SD and percentage change from the control diet unless stated otherwise. Data were analyzed using the PROC MIXED procedure for repeated measures in SAS with diet (MedDiet vs. control diet) as the main repeated effect (v9.2, Cary, NC). Individual response of HDL-C to MedDiet was heterogeneous and “responders” and “non-responders” to the MedDiet were identified based on an arbitrarily defined change in plasma HDL-C being positive (≥0.05 mmol/L) or negative (≤0.05 mmol/L). Subjects whose variations in HDL-C were close to 0 were excluded to maximize differences between groups. The two groups were compared using the non-parametric Wilcoxon-Mann–Whitney test while pair signed ranks were used to assess within-group changes. Pearson univariate correlation analyses adjusted for age were used to examine associations between diet-induced change in HDL-C and in other parameters. Variables with a skewed distribution were log-10 transformed prior to statistical analysis. Differences at *P* ≤ 0.05 (two-sided) were considered significant.

## Results

Characteristics at screening of the 26 participants with MetS are shown in Table 2[6]. Based on the food checklist, the mean compliance was 98.0 ± 5.3% for both isoenergetic experimental diets and was similar in both diets (not shown). The lipid profile and apoA-I kinetic data after the control diet and the MedDiet are presented in Table 3[6]. Body weight was reduced by 1.2 ± 0.9 kg (*P* < 0.001) despite efforts to keep participants in isoenergetic conditions. However, body weight was constant over the last three weeks of both isoenergetic diets (data not shown). The change in waist circumference did not quite reach statistical significance. Adjustment for the small change in body weight or waist circumference had no impact on the study outcomes (not shown). Plasma HDL-C concentrations as well as in HDL_2_ and HDL_3_ composition were similar after the MedDiet compared with the control diet. Consumption of the MedDiet led to significant reductions in plasma apoA-I concentrations and PS (both *P* ≤ 0.01) compared with the control diet. The MedDiet was also associated with a trend toward a reduction in apoA-I PR (*P* = 0.07), but had no impact on apoA-I FCR (*P* = 0.64) compared with the control diet.

**Table 2:** Physical characteristics and metabolic profile of the 26 male subjects at screening

**Variable**	**Mean ± SD**	**Range**	**Frequency of MetS criteria**
**Age (years)**	49.4 ± 11.6	24-62	-
**Weight (kg)**	98.3 ± 17.6	80.1-153.9	-
**Waist circumference (cm)**	110.9 ± 11.1	94.0-144.5	92.3%
**Systolic BP (mm Hg)**	123.8 ± 10.1	105-147	19.2%
**Diastolic BP (mm Hg)**	82.1 ± 6.6	71.5-94.5	42.3%
**Total-C (mmol/l)**	5.30 ± 1.22	2.46-7.88	-
**LDL-C (mmol/l)**	3.34 ± 1.05	1.36-6.07	-
**HDL-C (mmol/l)**	1.04 ± 0.29	0.34-1.90	46.2%
**Triglycerides (mmol/l)**	2.00 ± 0.82	0.52-3.71	57.7%
**Fasting glucose (mmol/l)**	5.66 ± 0.49	4.6-6.4	69.2%
**MetS (%)**	100	-	-

*BP* blood pressure, *C* cholesterol, *MetS* metabolic syndrome, Values are presented as mean ± SD. Criteria for MetS were: waist circumference ≥ 102 cm, systolic blood pressure ≥ 130 mm Hg or diastolic blood pressure ≥ 85 mm Hg, HDL-C <1.03 mmol/l, triglycerides ≥ 1.7 mmol/l and fasting glucose ≥ 5.6 mmol/l.

**Table 3:** **Lipid profiles and plasma apoA-I kinetics after the control diet and the MedDiet in 26 men with MetS**[6]

**Variables**	**Control diet**	**MedDiet**	**% change**	***P***^***a***^
Weight (kg) ^*b*^	98.4 ± 18.3	97.2 ± 18.3	−1.3%	<0.001
Waist circumference (cm) ^*b*^	111.5 ± 12.0	110.9 ± 11.7	−0.5%	0.056
VLDL-C (mmol/l) ^*b*^	0.43 ± 0.24	0.42 ± 0.19	−3.5%	0.762
VLDL-TG (mmol/l)	1.31 ± 0.55	1.30 ± 0.53	−0.3%	0.961
HDL-C (mmol/l) ^*b*^	0.91 ± 0.20	0.91 ± 0.19	0.0%	0.979
HDL_2_-C (mmol/l) ^*b*^	0.31 ± 0.10	0.31 ± 0.10	0.4%	0.829
HDL_3_-C (mmol/l)	0.61 ± 0.14	0.60 ± 0.14	−1.2%	0.642
HDL-TG (mmol/l)	0.14 ± 0.03	0.14 ± 0.03	−2.0%	0.645
HDL_2_-TG (mmol/l) ^*b*^	0.03 ± 0.01	0.03 ± 0.01	−3.8%	0.626
HDL_3_-TG (mmol/l) ^*b*^	0.11 ± 0.02	0.11 ± 0.02	−1.3%	0.773
HDL-ApoA-I (g/l)	1.10 ± 0.16	1.05 ± 0.16	−4.1%	0.014
Apo-AI				
Concentration (g/l)	1.24 ± 0.17	1.20 ± 0.16	−3.9%	0.010
PS (mg) ^*b*^	5521 ± 1341	5227 ± 1240	−5.3%	<0.001
PR (mg/kg/d)	17.8 ± 4.12	16.7 ± 2.97	−5.7%	0.073
FCR (pool/d)	0.32 ± 0.07	0.31 ± 0.06	−1.6%	0.642

*Apo* apolipoprotein, *C* cholesterol, *FCR* fractional catabolic rate, *HDL* high density lipoprotein, *PR* production rate, *PS* pool size, *TG* triglycerides, *VLDL* very low density lipoprotein. Values are presented as mean ± SD and percentage of change from values after the control diet.

^*a*^*P* value from the main effect of diet in the Mixed model (n = 26).

^*b*^ Analysis was performed on log-transformed values.

**Figure 2:**
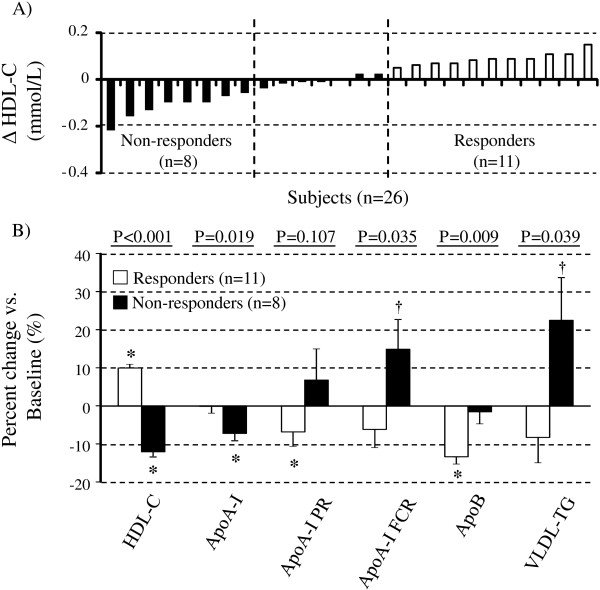
**Mean responses to the MedDiet in groups of HDL-C “responders” and “non-responders”.** Panel **A** Individual HDL-C responses to the MedDiet vs. control diet among the 26 men with MetS. Responders and non-responders of HDL-C were defined based on the change in plasma HDL-C concentrations being positive (≥0.05 mmol/L) or negative (≤0.05 mmol/L). Panel **B** Percent change from the control diet in metabolic variables according to changes in HDL-C with MedDiet (responders vs. non-responders). The *P* value for comparison between responders and non-responders is based on the Wilcoxon-Mann–Whitney test; *P* values for the change vs. control diet within groups is based on pair signed ranks test. Significant within-diet change from the control diet, *P < 0.05, †*P* < 0.11.

The individual HDL-C response to the MedDiet was highly heterogeneous (Figure 2, panel A). Participants among whom HDL-C concentrations were increased with MedDiet (responders: mean ∆HDL-C: 9.9 ± 3.2%, N = 11) showed significantly greater reductions in apoA-I FCR and in apoB and VLDL-TG concentrations (all *P* < 0.04) than those among whom HDL-C levels were reduced after the MedDiet (non-responders: mean ∆HDL-C: -12.0 ± 3.9%, N = 8) (Figure 2, panel B). Consumption of the MedDiet had no impact on plasma apoA-I concentrations among responders but significantly reduced apoA-I PR and apoB concentrations compared with the control diet (both *P* < 0.05). Among non-responders, there was a significant reduction in plasma apoA-I concentrations (*P* = 0.02) along with a trend toward an increase in apoA-I FCR and plasma VLDL-TG concentrations after MedDiet (both *P* < 0.11). There was no difference in age between responders and non-responders (data not shown). Diet-induced variations in HDL-C concentrations was significantly correlated with diet-induced variation in plasma apoA-I concentrations (*r* = 0.52), apoA-I FCR (*r* = −0.48) and VLDL-TG concentrations (*r* = −0.45, all *P* < 0.03). No correlation was observed between MedDiet-induced variations in HDL-C concentrations and apoA-I PR.

## Discussion

Men with MetS consumed a pre-determined MedDiet under carefully controlled isoenergetic feeding conditions, after standardization of the participants’ diet on a control diet to minimize inter-individual variations in baseline apoA-I kinetics. We showed that 4–5 week short-term consumption of a MedDiet significantly reduced plasma apoA-I concentrations and pool size, but had no impact on average on plasma HDL-C concentrations. This is at odds with data from studies having shown that adherence to MedDiet principles was associated with improvements in HDL-C concentrations [15,16]. However, adherence to MedDiet has also been associated with significantly lower body weight [15,16], which is likely to have confounded the effect of the diet on HDL-C concentrations [17,18]. Although consumption of the MedDiet had no impact on mean apoA-I FCR and plasma VLDL-TG concentrations, the individual HDL-C response to MedDiet in men with MetS appeared to be primarily determined by how apoA-I FCR and VLDL-TG concentrations were modified by the diet in each individual.

Consumption of the MedDiet vs. the control diet implied several changes in diet composition, including greater intakes of fibers, alcohol and MUFA and lower intakes of *trans* fatty acids (TFA) and SFA. Kinetic studies have shown that total dietary fat and/or SFA are associated with apoA-I PR (positively) and apoA-I FCR (negatively) [19,20]. A high MUFA diet consumed *ad libitum* reduced apoA-I PS with no significant change in apoA-I PR and FCR [21]. Consumption of *trans* fat has been shown to increase apoA-I FCR relative to a SFA rich diet in hypercholesterolemic women [22]. Water-soluble fibers have been shown to reduce LDL-C without affecting HDL-C concentrations [23]. Kinetic studies indicated that alcohol consumption increases plasma HDL-C and apoA-I concentrations mainly by increasing the PR of apoA-I [24,25]. Thus, variations in apoA-I kinetics in response to MedDiet in the present study must be interpreted in light of all of these individual nutrient-specific effects combined together. We hypothesize that the apparent reduction in apoA-I PR is partly attributable to the reduced amount of dietary SFA (−6.3%) in MedDiet vs. the control diet. Indeed, restricting dietary total fat and SFA has been shown to reduce hepatic apoA-I mRNA expression in livers of Cebus monkeys [20,26]. The significant reduction in LDL-C and apoB concentrations with MedDiet [6] may also have contributed to lowering apoA-I PR. Indeed, apoA-I PR has been positively correlated with plasma LDL-C and LDL-apoB concentrations [27], suggesting less need for reverse cholesterol transport when the plasma LDL-C pool is reduced. It appears that the impact of increasing alcohol intake as part of the MedDiet on raising apoA-I PR did not fully compensate for these effects.

Men with MetS in the present study were characterized by an elevated apoA-I FCR after the control diet (0.32 pool/day), and these figures are comparable with those from a previous kinetic study in which dyslipidemic subjects with MetS also had higher apoA-I FCR compared with controls (0.30 vs. 0.20 pool/day) [28]. Two other groups have shown that low HDL-C and apoA-I concentrations in overweight/obese subjects with insulin resistance were mainly accounted for by an apoA-I hypercatabolism [29,30]. Our results showed that the HDL-C response to MedDiet was highly heterogeneous. Participants among whom HDL-C increased with MedDiet showed greater reductions in apoA-I clearance rates and in plasma apoB and VLDL-TG concentrations than those among whom HDL-C concentrations were reduced with MedDiet. Moreover, correlation analysis showed that individual variations in the catabolism of apoA-I and in VLDL-TG concentrations were the strongest correlates of individual changes in HDL-C concentrations with MedDiet. Our data reaffirm that even in the context of significant dietary changes, the FCR of apoA-I remains the key determinant of the HDL-C and apoA-I response to MedDiet among men with MetS [2]. Indeed, although plasma apoA-I concentrations may be partly determined by the PR of apoA-I, change in the PR of apoA-I with MedDiet was not a significant correlate of concurrent variations in plasma concentrations of HDL-C and apoA-I in our study.

Several previous studies have shown that TG concentrations correlate positively with the catabolism of apoA-I [31,32]. Our data are consistent with that concept. Reduction in VLDL-TG decreases the hetero-exchange of neutral lipids by CETP leading to less TG-enriched HDL particles [33]. TG-poor HDL have been shown to be more stable and consequently, cleared less rapidly from the circulation [34]. We hypothesize that the increase in alcohol consumption with the MedDiet may be partly responsible for the heterogeneous TG response in these subjects with MetS. Indeed, a recent study has shown that heavy alcohol consumption can lead to either high or low concentrations of VLDL-TG [35]. Finally, low-carbohydrate/high-fat diets have HDL-C raising and TG lowering effects compared with high-carbohydrate/low-fat diets [36]. It is possible that the relatively high carbohydrate content of the MedDiet in our study may have attenuated its impact on plasma HDL-C concentrations. Indeed, a high fat MedDiet supplemented with nuts have been shown to reduce TG and increase HDL-C concentrations compared with a low fat diet [37].

To the best of our knowledge, this is the first study having documented the impact of the MedDiet on apoA-I kinetic in men with MetS. The carefully controlled feeding feature of the present study, the high compliance to the pre-determined diets and the relatively large number of participants considering a kinetic study are important strengths that need to be emphasized. Limitations of the current study pertain to the fact that there was no control group independent of the intervention and that participants were not randomized to the two experimental diets in this fixed sequence study. However, standardization of the baseline diet with a control North American diet prior to consuming the MedDiet allowed us to minimize inter-individual variations in baseline apoA-I kinetics and each participant acts as their own control. The sort-term duration of the study precludes any formal interpretation regarding longer term effects of MedDiet on HDL and apoA-I kinetics. Although MedDiet had no impact on HDL-C, some functions of HDL particles might still be beneficially altered by the diet, but this remained to be investigated.

## Conclusions

Data from this controlled feeding study suggest that the heterogeneous HDL-C response to a traditional MedDiet in men with MetS, independent of weight change, appears to be primarily determined by individual responses in apoA-I FCR and TG concentrations. The reduction in apoA-I PR with MedDiet apparently has no incidence on the HDL-C response to the diet and is probably due to the reduced amount of SFA and the concurrent reduction in LDL-C concentrations.

## Abbreviations

apoA-I: Apolipoprotein A-I; C: Cholesterol; CHD: Coronary heart disease; FCR: Fractional catabolic rate; HDL: High density lipoprotein; IEF: Isoelectric focusing; LDL: Low density lipoprotein; MedDiet: Mediterranean diet; MetS: Metabolic syndrome; MUFA: Monounsaturated fatty acids; PR: Production rate; PS: Pool size; PUFA: Polyunsaturated fatty acids; SFA: Saturated fatty acids; TFA: *Trans* fatty acids; TG: Triglycerides; VLDL: Very high density lipoprotein.

## Competing interests

The authors declare that they have no competing interests.

## Authors’ contributions

BL, PC, AHL and SD have designed and obtained funding for this study. PC was responsible for the screening and medical supervision of the study participants. CR coordinated the clinical study and performed statistical analyses, analyzed the data and wrote the manuscript, which was reviewed critically by all authors. BL is a Canada Research Chair in Nutrition and Cardiovascular Health. SD is a Canadian Institutes of Health Research (CIHR) New Investigator and a Fonds de la recherche en santé du Québec (FRSQ) Junior 1 Scholar. CR is recipient of doctoral scholarships from CIHR and FRSQ. All authors read and approved the final manuscript.
